# Mechanisms of Berberine for the Treatment of Atherosclerosis Based on Network Pharmacology

**DOI:** 10.1155/2020/3568756

**Published:** 2020-03-19

**Authors:** Xuejiao Xie, Xingyu Ma, Siyu Zeng, Wansi Tang, Liucheng Xiao, Chenggong Zhu, Rong Yu

**Affiliations:** ^1^Department of Zhongjing Theory, College of Chinese Medicine, Hunan University of Chinese Medicine, Changsha 410208, China; ^2^College of Chinese Medicine, Hunan University of Chinese Medicine, Changsha 410208, China

## Abstract

Atherosclerosis is a common metabolic disease characterized by lipid metabolic disorder. The processes of atherosclerosis include endothelial dysfunction, new endothelial layer formation, lipid sediment, foam cell formation, plaque formation, and plaque burst. Owing to the adverse effects of first-line medications, it is urgent to discover new medications to deal with atherosclerosis. Berberine is one of the most promising natural products derived from traditional Chinese medicine. However, the panoramic mechanism of berberine against atherosclerosis has not been discovered clearly. In this study, we used network pharmacology to investigate the interaction between berberine and atherosclerosis. We identified potential targets related to berberine and atherosclerosis from several databases. A total of 31 and 331 putative targets for berberine and atherosclerosis were identified, respectively. Then, we constructed berberine and atherosclerosis targets with PPI data. Berberine targets network with PPI data had 3204 nodes and 79437 edges. Atherosclerosis targets network with PPI data had 5451 nodes and 130891 edges. Furthermore, we merged the two PPI networks and obtained the core PPI network from the merged PPI network. The core PPI network had 132 nodes and 3339 edges. At last, we performed functional enrichment analyses including GO and KEGG pathway analysis in David database. GO analysis indicated that the biological processes were correlated with G1/S transition of mitotic cells cycle. KEGG pathway analysis found that the pathways directly associated with berberine against atherosclerosis were cell cycle, ubiquitin mediated proteolysis, MAPK signaling pathway, and PI3K-Akt signaling pathway. After combining the results in context with the available treatments for atherosclerosis, we considered that berberine inhibited inflammation and cell proliferation in the treatment of atherosclerosis. Our study provided a valid theoretical foundation for future research.

## 1. Introduction

Atherosclerosis is a common chronic disorder that plaque builds up in the arteries [1]. Plaque consists of cholesterol, calcium, fat, and other substances in the bloodstream [2]. High blood pressure, smoking, abnormal cholesterol levels, and obesity are considered as risk factors of atherosclerosis. Almost all people might suffer from atherosclerosis over the age of 65. Previous studies have demonstrated that several cardiovascular and cerebrovascular diseases such as coronary artery disease, cerebral infarction, cerebral hemorrhage were correlated with atherosclerosis [3, 4]. So, atherosclerosis is one of the most important factors leading to disability and death. This disorder leads to the heavy economic and social burden of society in the world [5]. Plaque formation is a slow process over several years with complex cellular and molecular mechanism [6]. In the early stage, circulating monocytes in the blood adhere to the endothelium and migrate into the subendothelial space [7]. Macrophages would be activated and oxidized lipoprotein particles are deposited under endothelial cells. Later, an inflammatory response cascade occurs as a result of endothelium damage. Increased production of proinflammatory mediators including interferons (IFNs), interleukins (ILs), transforming growth factors (TGFs), and tumor necrosis factors (TNFs) take part in the initiation and development of atherosclerosis [8]. The available treatments of atherosclerosis include statins, surgery, and other medications. However, there are many side effects of these methods [9]. Therefore, it is urgent to discover new medications to deal with atherosclerosis.

**Figure 1:**
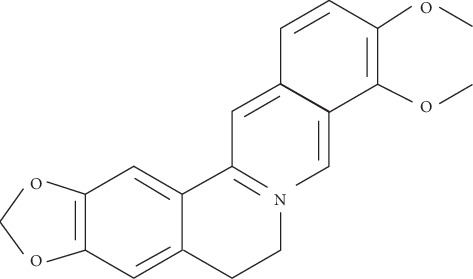
Chemical structure of berberine.

Berberine is a bioactive ingredient discovered in many plants such as Chinese goldthread, goldenseal, European barberry, tree turmeric, and Phellodendron [10].  Figure 1 showed the chemical structure of berberine.

Traditional Chinese medicine takes into account that berberine has excellent effects including resolving dampness, clearing away heat, detoxification, and purging fire. In addition, berberine is the main ingredient in the many famous decoctions of Chinese medicine. The famous decoctions Gegen-Huangqin-Huanglian decoction (*Coptidis rhizome*, *Scutellariae radix*, *Puerariae lobatae radix*, *Glycyrrhizae radix*) and Huanglian-Jie-Du decoction (*Scutellariae radix*, *Coptidis rhizome*, *Gardeniae fructus*, *Phellodendri cortex*) contain berberine [11, 12]. Previous studies have revealed that berberine was beneficial in treating many disorders. Lin et al. found that berberine was effective in inhibiting cell proliferation and promoting apoptosis of breast cancer cells [13]. Jin et al. displayed that berberine inhibited osteosarcoma by suppressing the caspase-1/IL-1*β* signaling axis [14]. Ma et al. summarized that berberine alleviated diabetes mellitus by combating inflammation and oxidative stress [15]. Except for cancers and metabolic diseases, berberine also had therapeutic effects in atherosclerosis [16]. Berberine exerted protective effects against atherosclerosis by regulating various proatherogenic cellular and molecular mechanisms. Endothelial functions, vascular smooth muscle cells migration, macrophage-derived foam formation, and platelet activation might be involved in the protective effects of berberine against atherosclerosis. However, the molecular targets and mechanisms of berberine in dealing with atherosclerosis have not been explored clearly.

Network pharmacology is a developing area based on systemic pharmacology to investigate the interaction between drugs and diseases [17]. In the network pharmacology, the drug-target network is important to understand the mechanism of drugs. The disease targets could be applied in the field of drug design, drug discovery, and biomarkers detection [18]. Complex diseases such as cardiovascular diseases and cancers do not attribute to single-molecule mutation or signaling pathway dysfunction. They are usually caused by the whole regulation of network dysfunction. Network pharmacology could lead to the progression of methodology and theory in drug design.

In the present study, we explored the mechanism of berberine in treating atherosclerosis with network pharmacology. We identified potential targets related to berberine and atherosclerosis. Then, we constructed protein-protein interaction (PPI) networks of berberine targets and atherosclerosis targets. Later, we merged the two PPI networks and analyzed the network topological features to obtain the core PPI network. At last, we performed the functional enrichment analyses including Gene Ontology (GO) and Kyoto Encyclopedia of Genes and Genomes (KEGG) signaling pathway analysis to characterize the biological processes and pathways. The results of this study provided a comprehensive and meaningful analysis of berberine in treating atherosclerosis. This study also provided a novel mechanism of berberine against atherosclerosis.

## 2. Methods

### 2.1. Screening of Potential Targets for Berberine

Two databases were conducted to identify potential targets related to berberine. The two databases included traditional Chinese medicine systems pharmacology (TCMSP) database and STITCH database. TCMSP database (http://lsp.nwu.edu.cn/tcmsp.php) is a unique system pharmacological database of traditional Chinese medicines describing the relationships between drugs, targets, and diseases. STITCH database (http://stitch.embl.de/) is a useful tool to discover the interaction networks of chemicals and proteins.

### 2.2. Screening of Potential Targets for AS

Four databases were applied to identify potential targets related to atherosclerosis. The four databases included Therapeutic Target Database (TTD), DrugBank database, Genetic Association Database (GAD), and Online Mendelian Inheritance in Man (OMIM). TTD (https://db.idrblab.org/ttd/) is a useful database providing data about exploring therapeutic protein and nucleic acid targets, the targeted disease. The DrugBank database (https://www.drugbank.ca/) is a powerful and comprehensive bioinformatics database containing information on drugs and disease. GAD database (https://geneticassociationdb.nih.gov/) is a useful database of genetic and protein association data from complex disorders and diseases. OMIM (https://www.omim.org/) is an authoritative, timely, and comprehensive database about human genes and genetic diseases.

### 2.3. Construction of PPI Networks

In this study, we acquired PPI data from the Cytoscape plugin BisoGenet. Six PPI databases in the Cytoscape plugin BisoGenet included the Database of Interacting Proteins (DIP), the Biological General Repository for Interaction Datasets (BioGRID), the Human Protein Reference Database (HPRD), the IntAct Molecular Interaction Database (IntAct), the Molecular INTeraction Database (MINT), and the Biomolecular Interaction Network Database (BIND). PPI network predicting berberine targets was visualized and constructed by Cytoscape software (version 3.7.1, Boston, MA, USA). In addition, the PPI network predicting atherosclerosis targets was visualized and constructed by Cytoscape software, either. After obtaining two PPI networks, we merged these two networks to achieve the core PPI network. Later, topological features of the core PPI network were analyzed.

### 2.4. Analysis of Network Topological Features

Six parameters were used to analyze the topological properties of every node in the interaction network. The six parameters included degree centrality (DC), betweenness centrality (BC), closeness centrality (CC), eigenvector centrality (EC), local average connectivity (LAC), and network centrality (NC). The plugin cytoNCA in the Cytoscape software was conducted to perform the analysis. The computation equations and definitions of these parameters indicated the topological importance of nodes in the core PPI network.

### 2.5. Functional Enrichment Analyses

The Database for Annotation, Visualization, and Integrated Discovery (David) was used to perform GO and KEGG pathway analyses in this study. GO analysis is a useful bioinformatics tool to characterize molecular function (MF), cellular components (CC), and biological process (BP) of genes. KEGG pathway analysis is a collection of databases describing biological pathways, genomes, drugs, and diseases. The OmicShare platform (https://www.omicshare.com/) was used to visualize the bubble chart.

## 3. Results

### 3.1. Potential Targets of Berberine

**Figure 2:**
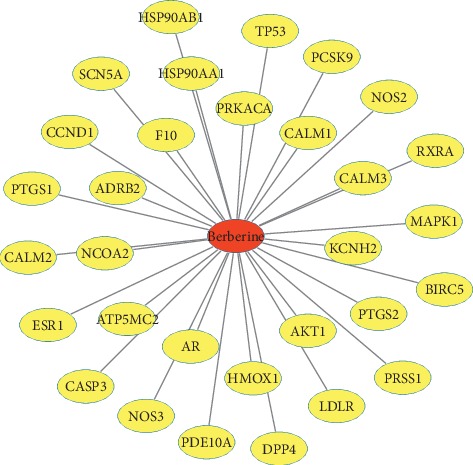
Potential targets of berberine. Red node represented berberine. Yellow nodes represented the targets of berberine.

A total of 31 putative targets of berberine were identified with TCMSP and STITCH databases. The putative targets included ADRB2, CALM1, HSP90AA1, KCNH2, and so on. Detailed information about potential targets of berberine was listed in Figure 2 and Supplementary [Supplementary-material supplementary-material-1].

### 3.2. Potential Targets of Atherosclerosis

**Figure 3:**
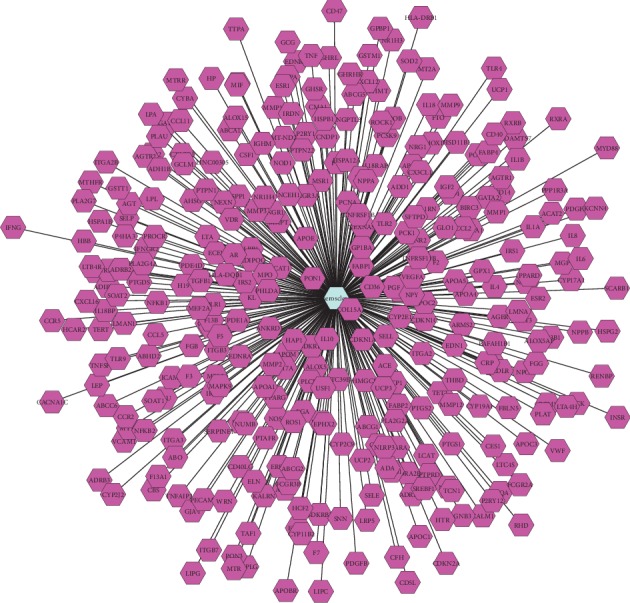
Potential targets of atherosclerosis. Blue node represented atherosclerosis. Purple nodes represented targets of atherosclerosis.

A total of 331 putative targets of atherosclerosis were identified with TTD, DrugBank, GAD, OMIM databases. The putative targets included ABCA1, ANKRD1, CNR2, ICAM1, and so on. The detailed information about the potential targets of atherosclerosis was listed in Figure 3 and Supplementary [Supplementary-material supplementary-material-1].

### 3.3. PPI Network Construction

**Figure 4:**
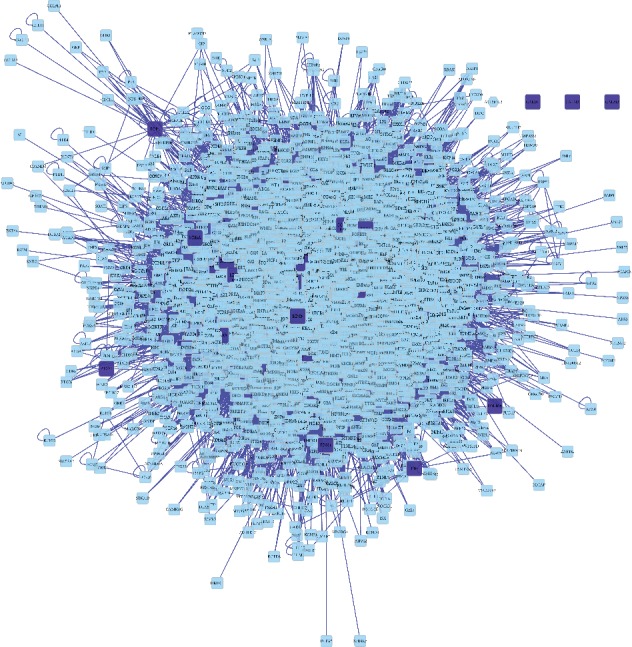
PPI network of berberine targets. Dark blue square nodes represented the targets of berberine. Light blue square nodes represented the proteins interacting with the targets of berberine. The edges represented the relationship between them.

PPI networks were used to understand the functions of diverse targets in berberine and atherosclerosis. In this study, we constructed a berberine targets network with PPI data (Figure 4). The results showed that berberine targets PPI network had 3204 nodes and 79437 edges.

**Figure 5:**
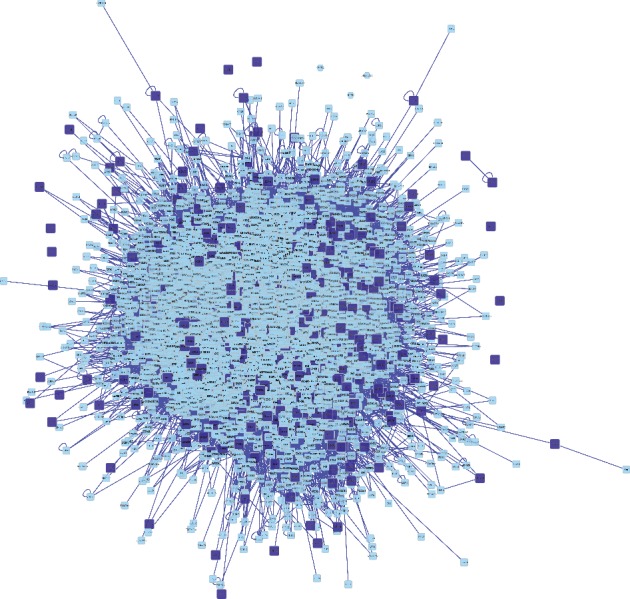
PPI network of atherosclerosis targets. Dark blue square nodes represented the targets of atherosclerosis. Light blue square nodes represented the proteins interacting with the targets of atherosclerosis. The edges represented the relationship between them.

In addition, we constructed atherosclerosis targets network with PPI data (Figure 5). The results indicated that atherosclerosis targets PPI network had 5451 nodes and 130891 edges.

**Figure 6:**
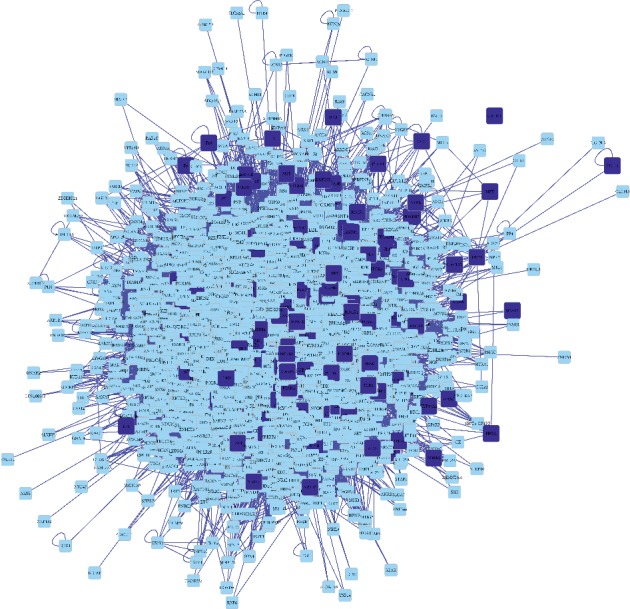
Merged PPI network of berberine targets PPI network and atherosclerosis targets PPI network. Dark blue square nodes represented the targets of berberine and atherosclerosis. Light blue square nodes represented the proteins interacting with the targets of berberine and atherosclerosis. The edges represented the relationship between them.

Furthermore, we used the merge network function to construct a new PPI network in order to discover the pharmacological mechanism of berberine against atherosclerosis. The merged PPI network was constructed in Cytoscape software. The results showed that the merged PPI network had 2393 nodes and 67362 edges (Figure 6).

### 3.4. Network Topological Features Analysis

**Figure 7:**
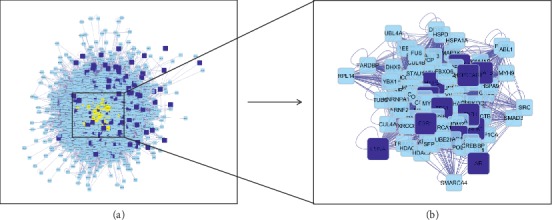
Core PPI network under network topological features analysis. (a) Six parameters were used to analyze the topological properties of the merged PPI network. The parameters were listed as follows: DC > 71，BC > 0.00074, CC > 0.4829, EC > 0.0301, NC＞30.53, LAC > 20.34. (b) Core PPI network.

In order to obtain a core PPI network from the merged PPI network, we conducted six parameters to analyze the topological properties of the merged PPI network. The six parameters contained DC, BC, CC, EC, LAC, and NC. The parameters were listed as follows: DC > 71, BC > 0.00074, CC > 0.4829, EC > 0.0301, NC > 30.53, LAC > 20.34.  Figure 7 displayed the core PPI network.

The core PPI network had 132 nodes and 3339 edges. The nodes in the core PPI network included neurotrophic receptor tyrosine kinase 1 (NTRK1), estrogen receptor 1 (ESR1), tumor protein p53 (TP53), cullin 3 (CUL3), heat shock protein 90 (HSP90), amyloid beta precursor protein (APP), minichromosome maintenance complex component 2 (MCM2), fibronectin 1 (FN1), ubiquitin C (UBC), and so on.

### 3.5. Functional Enrichment Analyses

**Figure 8:**
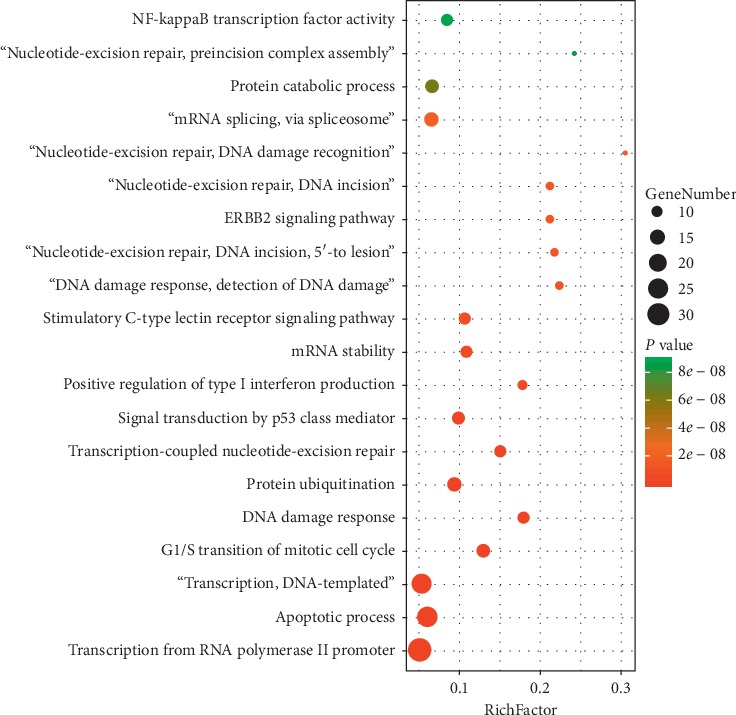
GO analysis of candidate targets for berberine against atherosclerosis. The *X*-axis showed the enrichment scores of these terms, and the *Y*-axis showed significantly enriched GO categories of the target genes. The size of the dots represented the counts of the genes. The color of the dots represented the *P*-value.

David was used to perform GO and KEGG pathway analysis in this study. GO analysis of candidate targets for berberine against atherosclerosis was conducted to discover the biological process in David database. The results showed that the biological processes were correlated with the regulation of transcription from RNA polymerase II promoter, negative regulation of the apoptotic process, regulation of DNA-templated transcription, G1/S transition of the mitotic cell cycle, DNA damage response, protein ubiquitination, transcription-coupled nucleotide-excision repair, nucleotide-excision repair (Figure 8). The target genes of each biological process were list in Supplementary [Supplementary-material supplementary-material-1].

**Figure 9:**
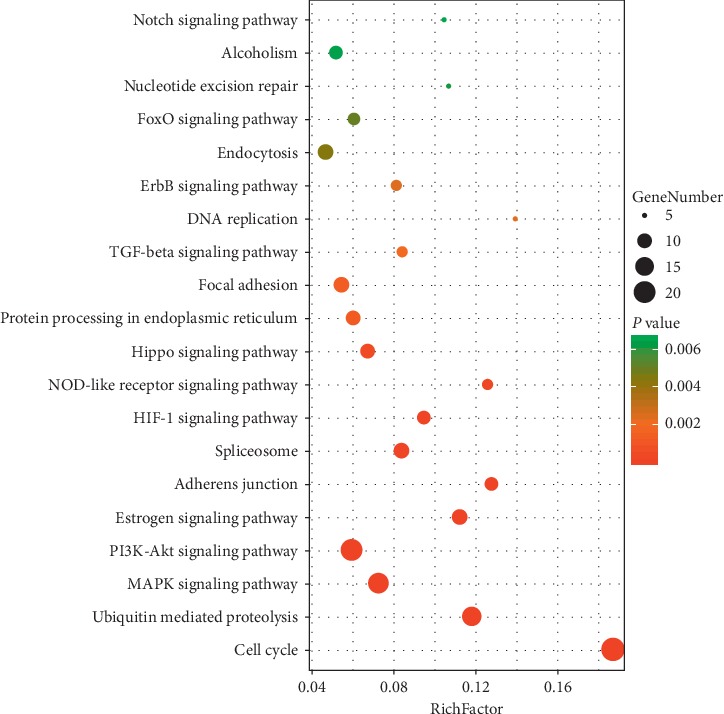
KEGG pathway analysis of candidate targets for berberine against atherosclerosis. The *X*-axis showed the enrichment scores of these terms, and the *Y*-axis showed significantly enriched KEGG categories of the target genes. The size of the dots represented the counts of the genes. The color of the dots represented the *P*-value.

Besides, we conducted a KEGG pathway analysis to investigate biological pathways of berberine against atherosclerosis. The top 10 signaling pathways were listed as follows: cell cycle, ubiquitin mediated proteolysis, MAPK signaling pathway, PI3K-Akt signaling pathway, Estrogen signaling pathway, adherens junction, spliceosome, HIF-1 signaling pathway, NOD-like receptor signaling pathway, and Hippo signaling pathway (Figure 9). The target genes of each pathway were list in Supplementary [Supplementary-material supplementary-material-1].

## 4. Discussion

Atherosclerosis is a common metabolic disease characterized by lipid metabolic disorder. It is a multifactorial pathological disorder changing the morphology and function of arterial walls [19, 20]. Statins are the first-line lipid-lowering medications to deal with atherosclerosis [21]. Statins also lead to many side effects in some population [22]. Except for statins, other lipid-regulating medications also have some side effects. So, it is important to discover new methods to treat atherosclerosis.

As a member of traditional medicine, berberine is widely used in Asian countries such as China, Korea, and Japan owing to the clinical and safety profiles [23]. Previous studies have demonstrated the therapeutic potential of berberine inhibiting atherosclerosis *in vivo*. Berberine administration significantly decreased the atherosclerotic plaque area in ApoE^−/−^ mice [24]. In homocysteine thiolactone (HTL)-fed ApoE^−/−^ mice, berberine promoted the stability of plaque in the artery by downregulating the oxidative stress [25]. However, the mechanism of berberine against atherosclerosis has not been discovered clearly. Network pharmacology is a new area to investigate the interaction between drugs and diseases. It could provide the whole picture of drug mechanisms in dealing with the disease. In this study, we constructed berberine and atherosclerosis targets networks with PPI data. Then, we merged the two PPI networks and analyzed the topological properties of the merged PPI network to obtain the core PPI network. Furthermore, we performed functional enrichment analyses, including GO and KEGG pathway analysis, to clarify the multiple mechanisms of berberine against atherosclerosis.

Current treatments of atherosclerosis aim to control risk factors and to maintain perfusion in affected arteries. The available drugs to deal with atherosclerosis include cholesterol medication, antiplatelet medication, and other medications [26]. Cholesterol medication statins and antiplatelet mediation aspirin are widely used as established interventions in the treatment of atherosclerosis. The available treatments of atherosclerosis possess pleiotropic aspects of mechanisms to deal with atherosclerosis. According to our results in this study, berberine might possess a similar mechanism with previous drugs against atherosclerosis.

Firstly, available treatment statins and aspirin alleviate the inflammatory status of atherosclerosis. Atherosclerosis is a chronic inflammatory disease, and inflammation plays a critical role in the pathogenesis of atherosclerosis [27]. In the inflammatory response of blood vessels, inflammatory cells infiltrate vascular endothelium and release inflammatory mediators to exacerbate atherosclerosis [28]. Several inflammatory signaling pathways including the MAPK signaling pathway, PI3K-Akt signaling pathway, and ubiquitin mediated proteolysis are involved in the inflammatory response to various intracellular and extracellular stimuli [29, 30]. The available treatment statins and aspirin beneficially affect atherogenesis and prevent the progression of atherosclerosis [31]. Statins have been revealed to regulate intracellular inflammatory pathways. The expression of inflammatory cytokines including TNF-*α*, IL-6, and IFN-*γ* produced by SMCs and macrophages could be downregulated by statins [32]. Apart from inflammatory cytokines, statins inhibit the chemokines expression such as monocyte chemoattractant protein-1 (MCP-1) to reduce inflammatory cell infiltration. C-reactive protein (CRP), a biomarker of inflammation, could be downregulated after statins treatment. Previous studies also discovered that long-term aspirin administration decreased aortic atherosclerotic injury and vascular inflammation [33]. Vascular inflammatory signs including TNF-*α*, MCP-1, and NF-*κ*B activity were inhibited by aspirin treatment. The endothelial function in the presence of inflammation could be protected by aspirin. In our study, we revealed that the beneficial effects of berberine in atherosclerosis are attributed to their anti-inflammatory properties. KEGG analysis in our study indicated that signaling pathways of berberine against atherosclerosis were related to ubiquitin mediated proteolysis, MAPK signaling pathway, and PI3K-Akt signaling pathway. These inflammatory processes take part in the different stages of atherosclerosis. Ubiquitin-proteasome system could regulate many biological cellular processes such as inflammation and oxidative stress [34]. Dysfunction of the ubiquitin-proteasome system plays a crucial role in the plaque progression and tendency to rupture of plaque. The GO analysis also discovered that protein ubiquitination involved in ubiquitin-dependent protein catabolic process was correlated with the effects of berberine against atherosclerosis. Inflammatory MAPK signaling pathway plays an important role in the pathogenesis of foam cell formation in atherosclerosis [35]. Foam cell formation was dependent on the activation of components of MAPK inflammatory pathways such as JNK2 and p38*α* MAPK. PI3K-Akt signaling pathway could regulate inflammatory processes of atherosclerosis including cellular composition and final fibrous cap establishment [30]. Inhibition of PI3K-Akt signaling pathway could enhance the stability of atherosclerosis plaques. Similar to the anti-inflammatory effects of available treatments, we inferred that berberine possessed anti-inflammatory effects by inhibiting signaling pathways in treating atherosclerosis.

Except for inhibiting inflammation, available treatments such as statins inhibit cell proliferation in the treatment of atherosclerosis [36]. The proliferation of arterial smooth muscle cells (SMCs) contributes to the plaque fibrous cap formation and inflammatory cytokines production. Inhibition of SMCs proliferation by statins delays the plaque progression [37]. In our study, we found that the cell cycle was involved in the mechanism of berberine against atherosclerosis in KEGG analysis. In addition, GO analysis showed G1/S transition of mitotic cells cycle was correlated with the biological process of berberine against atherosclerosis. G1/S transition of mitotic cells cycle takes part in the mechanism of the cell cycle [38]. So, we inferred berberine might inhibit the proliferation of SMCs by influencing the cell cycle.

Taken together, the available treatments such as aspirin and statins deal with atherosclerosis by inhibiting inflammation and cell proliferation. After combining the results in context with the available treatments for atherosclerosis, we considered that berberine inhibits inflammation and cell proliferation in the treatment of atherosclerosis. The inflammatory pathways including ubiquitin mediated proteolysis, MAPK signaling pathway, and PI3K-Akt signaling pathway could be suppressed by berberine. Aberrant cell proliferation could be attenuated by barbering by regulating the cell cycle. G1/S transition of mitotic cells cycle might be involved in the mechanism of berberine in regulating cell cycle.

## 5. Conclusion

In conclusion, the multitargets and multicomponents of berberine effects against atherosclerosis were clearly clarified with network pharmacology. We identified potential targets related to berberine and atherosclerosis with several databases. Then, we merged the two PPI networks and obtained the core PPI network from the merged PPI network. At last, we performed functional enrichment analyses including GO and KEGG pathway analysis to clarify the multiple mechanisms of berberine against atherosclerosis. The pathways directly associated with berberine against atherosclerosis were cell cycle, ubiquitin mediated proteolysis, MAPK signaling pathway, and PI3K-Akt signaling pathway. After combining the results in context with the available treatments for atherosclerosis, we considered that berberine could inhibit inflammation and cell proliferation in the treatment of atherosclerosis.

## Data Availability

The data used to support the findings of this study are included within the article.

## Abbreviations

APP: Amyloid beta precursor protein
BC: Betweenness centrality
BIND: Biomolecular Interaction Network Database
BioGRID: Biological General Repository for Interaction Datasets
BP: Biological process
CC: Closeness centrality
CRP: C-reactive protein
CUL3: cullin 3
David: Database for Annotation, Visualization and Integrated Discovery
DC: Degree centrality
DIP: Database of Interacting Proteins
EC: Eigenvector centrality; ESR1, estrogen receptor 1
FN1: Fibronectin 1
GAD: Genetic Association Database; GO, Gene Ontology
HIF-1: Hypoxia-inducible factor 1
HPRD: Human Protein Reference Database
HSP90: Heat shock protein 90
ICAM-1: Intercellular adhesion molecule 1
IFNs: Interferons
ILs: Interleukins
iNOS: Inducible nitric oxide synthase
IntAct: IntAct Molecular Interaction Database
KEGG: Kyoto Encyclopedia of Genes and Genomes
LAC: local average connectivity
MAPK: Mitogen-activated protein kinase
MCM2: Minichromosome maintenance complex component 2
MCP-1: Chemoattractant protein-1
MF: Molecular function
MINT: Molecular INTeraction Database
MMP: matrix metalloproteinase
NC: Network centrality
NF-κB: Nuclear factor kappa B
NO: Nitric oxide
NTRK1: Neurotrophic receptor tyrosine kinase 1
OMIM: Online Mendelian Inheritance in Man
OxLDL: Oxidized low density lipoprotein
ROS: Reactive oxygen species
PI3K: Phosphoinositide 3-kinase
PPI: Protein-protein interaction
SMCs: smooth muscle cells
TCMSP: Traditional Chinese medicine systems pharmacology
TGFs: Transforming growth factors
TNFs: Tumor necrosis factors
TP53: Tumor protein p53
TTD: Therapeutic Target Database
UBC: Ubiquitin C
VSMCs: Vascular smooth muscle cells

## Supplementary Materials

Supplementary Materials Supplementary Table 1: potential targets of berberine. Supplementary Table 2: potential targets of atherosclerosis. Supplementary Table 3: target genes of each biological process. Supplementary Table 4: target genes of each pathway.
