# Supplementary material for: Mechanisms of Berberine for the Treatment of Atherosclerosis Based on Network Pharmacology
Source: Evid Based Complement Alternat Med. 2020 Mar 19;2020:3568756. doi: 10.1155/2020/3568756 (PMC7106879; doi:10.1155/2020/3568756)
Supplement: Supplementary Materials — Supplementary Table 1: potential targets of berberine. Supplementary Table 2: potential targets of atherosclerosis. Supplementary Table 3: target genes of each biological process. Supplementary Table 4: target genes of each pathway. [file 3568756.f1.pdf]

Supplementary table 1. Potential targets of berberine.

Compound	Targets
Berberine	ADRB2, AR, CALM1, CALM2, CALM3, ESR1, F10, HSP90AA1, HSP90AB1, KCNH2, NCOA2, NOS2, NOS3, PDE10A, PRKACA, PRSS1, PTGS1, PTGS2, RXRA, SCN5A, BIRC5, AKT1, ATP5MC2, CASP3, CCND1, DPP4, HMOX1, LDLR, MAPK1, PCSK9, TP53

Supplementary table 2. Potential targets of atherosclerosis.

Disease	Targets
Atherosclerosis	ABCA1, ABCC6, ABCG1, ABCG2, ABCG5, ABHD2, ABO, ACAT1, ACAT2, ACE, ACP1, ADA, ADAMTS7, ADD1, ADH1B, ADH7, ADIPOQ, ADIPOR1, ADRA1B, ADRA2B, ADRB2, ADRB3, AGER, AGT, AGTR1, AGTR2, AHSG, ALOX15, ALOX5, ALOX5AP, ANGPTL3, ANKRD1, AP3B1, APOA1, APOA4, APOA5, APOB, APOBR, APOC1, APOC2, APOC3, APOE, APOL3, APOM, AR, ARMS2, BCHE, BDKRB1, BDKRB2, BHMT, BIRC3, BRD4, C1QA, CACNA1C, CALM1, CBS, CCL11, CCL2, CCL5, CCR2, CCR5, CD14, CD36, CD40, CD40LG, CD47, CD5L, CDKN1A, CDKN1C, CDKN2A, CES1, CETP, CFH, CLU, CMA1, CNDP1, CNR2, COL15A1, CREG1, CRP, CSF1, CST3, CX3CL1, CX3CR1, CXCL12, CXCL16, CYBA, CYP11B2, CYP17A1, CYP19A1, CYP2C9, CYP2E1, CYP2J2, CYP7A1, ECE1, EDN1, EDNRA, EDNRB, ELN, ENPP1, EPHX2, EREG, ESD, ESR1, ESR2, F12, F13A1, F13B, F2, F3, F5, F7, FABP1, FABP2, FABP4, FBLN5, FCGR2A, FCGR3A, FCGR3B, FGA, FGB, FGG, FTO, GATA2, GCG, GCK, GCLM, GDF15, GHRHR, GHRL, GHSR, GJA4, GLO1, GNB3, GP1BA, GPBP1, GPX1, GSTM1, GSTT1, H19, HAP1, HBB, HCAR2, HCF2, HLA-DQB1, HLA-DRB1, HMGCR, HMOX1, HP, HSD11B1, HSPA12A, HSPA12B, HSPA1B, HSPB1, HSPG2, HTR, ICAM1, IFNG, IFNGR1, IFNGR2, IGF2, IGHM, IL10, IL17A, IL18, IL18BP, IL18R1, IL18RAP, IL1A, IL1B, IL1RN, IL4, IL6, IL8, INS, INSR, IRDN, IRF1, IRS1, IRS2, ITGA2, ITGA2B, ITGA3, ITGB3, ITGB7, KALRN, KCNN4, KL, LCAT, LDLR, LEP, LGALS2, LINC00305, LIPC, LIPG, LMAN1, LMNA, LPA, LPL, LRP1, LRP5, LTA, LTA4H, LTB4R, LTC4S, MAPK9, MAPT, MBL2, MEF2A, MGP, MIF, MMP1, MMP12, MMP13, MMP2, MMP3, MMP9, MPO, MSR1, MT2A, MTHFR, MT-ND2, MTR, MTRR, MTTP, MYB, MYD88, NCEH1, NEU1, NEXN, NEXNAS1, NFKB1, NFKB2, NLRP3, NOD1, NOD2, NOS2, NOS3, NPC1, NPPA, NPPB, NPY, NR1H2, NR1H3, NR1H4, NR3C1, NRG1, NUMB, OLR1, P2RY1, P2RY12, P4HA3, PAFAH1B1, PAPPA, PCK1, PCNA, PCSK9, PDE1A, PDE4D, PDGFA, PDGFB, PECAM1, PGF, PGM1, PHLDA1, PLA2G2A, PLA2G4A, PLA2G7, PLAT, PLAU, PLCB3, PON1, PON2, PON3, PPARA, PPARD, PPARG, PPP1R3A, PROCR, PTAFR, PTGDS, PTGS1, PTGS2, PTPN1, PTPN22, PTPRD, RENBP, RHD, ROCK1, ROS1, RXRA, RXRB, SCARB1, SELE, SELL, SELP, SELPLG, SERPINE1, SFTPD, SNN, SOAT1, SOAT2, SOD2, SPP1, SREBF1, TAF1, TCN1, TERT, TET2, TGFB1, THBD, TLR2, TLR4, TLR9, TNF, TNFAIP3, TNFRSF11B, TNFRSF1A, TNFRSF1B, TNFSF4, TTC39B, TTPA, UCP1, UCP2, UCP3, USF1, VCAM1, VDR, VEGFA, VWF, WRN

Supplementary table 3. Target genes of each biological process.

Term	Genes

transcription from RNA polymerase II promoter	HNRNPK, SMAD3, NEDD8, NFKB1, SNW1, RB1, RUVBL1, CDC5L, DDX5, BRCA1, SMARCA4
apoptotic process	RACK1, MAPK1, CDK1, EP300, MAP3K1, IKBKG, TP53, NFKB1, MCM2, RB1, BRCA1, CUL1
transcription, DNA-templated	EEF1A1, AR, VHL, CREBBP, EZH2, TP53, ESR1, SMAD3, YBX1, POLR2A, EP300, HDAC1, TARDBP, HNRNPD, RUVBL2, RUVBL1, ABL1, MYC, EWSR1, SMARCA4
G1/S transition of mitotic cell cycle	CDK1, RB1, MCM2, CDK2, MCM5, CUL3, RPA1, CUL2, RPA2, CUL5, CUL4A, PCNA, CUL1
DNA damage response	CDK1, EP300, NPM1, PCNA, TP53, UBC, MDM2, AURKA, SFN, RPS27A, CDK2
protein ubiquitination	HSP90AB1, HSP90AA1, HSPA1A, HSPA1B, PARK2
transcription-coupled nucleotide-excision repair	RPA1, RPA2, EP300, CUL4A, COPS5, COPS6, PCNA, UBC, CUL4B, RPS27A, POLR2A
signal transduction by p53 class mediator	RPA1, RPA2, HDAC2, EP300, HDAC1, TP53, UBC, MDM2, AURKA, BRCA1, RPS27A, CDK2
positive regulation of type I interferon production	XRCC5, DHX9, EP300, RELA, XRCC6, CREBBP, PRKDC, NFKB1, CTNNB1
mRNA stability	XPO1, YWHAZ, PSMA3, HNRNPD, UBC, YWHAB, HSPB1, HSPA1A, HSPA1B, RPS27A, HSPA8
stimulatory C-type lectin receptor signaling pathway	EP300, RELA, PSMA3, CREBBP, IKBKG, UBC, NFKB1, TRAF6, RPS27A, SRC, CUL1
DNA damage response, detection of DNA damage	RPA1, RPA2, CUL4A, PCNA, UBC, CUL4B, PARP1, RPS27A
nucleotide-excision repair, DNA incision, 5'-to lesion	RPA1, RPA2, CUL4A, PCNA, UBC, CUL4B, PARP1, RPS27A
ERBB2 signaling pathway	EGFR, CUL5, HSP90AA1, GRB2, UBC, SHC1, RPS27A, SRC
nucleotide-excision repair, DNA incision	RPA1, RPA2, CUL4A, CUL4B, PARP1
nucleotide-excision repair, DNA damage recognition	CUL4A, COPS5, COPS6, UBC, CUL4B, PARP1, RPS27A
mRNA splicing, via spliceosome	FUS, DHX9, U2AF2, SNW1, CDC5L, DDX5, HNRNPA1, YBX1, HNRNPU, POLR2A, EIF4A3,

	HNRNPK, HNRNPD, HSPA8
protein catabolic process	CDK1, AURKA, PARK2, CUL3, CUL2, CUL5, HUWE1, CUL4A, UBC, MDM2, SMURF1, CUL4B, CUL1, RPS27A
nucleotide-excision repair, preincision complex assembly	RPA1, RPA2, CUL4A, UBC, CUL4B, PARP1, RPS27A
NF-kappaB transcription factor activity	AR, RELA, NTRK1, NPM1, IKBKG, UBC, NFKB1, HSPA1A, HSPA1B, TRAF6, RPS27A

Supplementary table 4. Target genes of each pathway.

**Term**	**Genes**
Cell cycle	CDK1, YWHAZ, CREBBP, TP53, YWHAB, SMAD3, PRKDC, RB1, MCM2, SFN, YWHAE, CDK2, MCM5, YWHAG, HDAC2, EP300, HDAC1, PCNA, YWHAQ, MDM2, ABL1, MYC, CUL1
Ubiquitin mediated proteolysis	VHL, UBE2I, PARK2, BRCA1, CUL3, CUL2, CUL5, CUL7, HUWE1, CUL4A, MAP3K1, MDM2, SMURF1, CUL4B, TRAF6, CUL1
MAPK signaling pathway	EGFR, GRB2, RELA, TP53, NFKB1, HSPA1A, HSPA1B, FLNA, MAPK1, MAP3K3, ARRB2, NTRK1, MAP3K1, IKBKG, HSPB1, TRAF6, MYC, HSPA8
PI3K-Akt signaling pathway	EGFR, HSP90AB1, YWHAZ, HSP90AA1, GRB2, RELA, TP53, YWHAB, NFKB1, ITGA4, YWHAE, BRCA1, CDK2, MAPK1, YWHAG, IKBKG, YWHAQ, MDM2, MYC, FN1
Estrogen signaling pathway	HSP90AB1, EGFR, MAPK1, HSP90AA1, GRB2, ESR1, SHC1, HSPA1A, HSPA1B, SRC, HSPA8
Adherens junction	ACTB, EGFR, MAPK1, EP300, CREBBP, SMAD3, SRC, IQGAP1, CTNNB1
Spliceosome	EIF4A3, HNRNPK, U2AF2, SNW1, HSPA1A, HSPA1B, CDC5L, DDX5, HNRNPA1, HNRNPU, HSPA8
HIF-1 signaling pathway	EGFR, MAPK1, CUL2, EP300, VHL, RELA, CREBBP, NFKB1, GAPDH
NOD-like receptor signaling pathway	HSP90AB1, MAPK1, HSP90AA1, RELA, IKBKG, NFKB1, TRAF6
Hippo signaling pathway	ACTB, PPP1CA, YWHAZ, YWHAG, YWHAB, YWHAQ, SMAD3, YWHAE, MYC, CTNNB1

Protein processing in endoplasmic reticulum	HSP90AB1, HSP90AA1, VCP, FBXO6, HSPA1A, HSPA1B, HSPA5, PARK2, HSPA8, CUL1
Focal adhesion	ACTB, EGFR, MAPK1, PPP1CA, GRB2, SHC1, ITGA4, FLNA, SRC, CTNNB1, FN1
TGF-beta signaling pathway	MAPK1, EP300, CREBBP, SMAD3, SMURF1, MYC, CUL1
DNA replication	RPA1, RPA2, PCNA, MCM2, MCM5
ErbB signaling pathway	EGFR, MAPK1, GRB2, SHC1, ABL1, MYC, SRC
Endocytosis	EGFR, ARRB2, SMAD3, MDM2, HSPA1A, HSPA1B, SMURF1, CLTC, TRAF6, SRC, HSPA8
FoxO signaling pathway	EGFR, MAPK1, EP300, GRB2, CREBBP, SMAD3, MDM2, CDK2
Nucleotide excision repair	RPA1, RPA2, CUL4A, PCNA, CUL4B
Alcoholism	HDAC5, MAPK1, PPP1CA, HDAC3, HDAC2, HDAC1, GRB2, H2AFX, SHC1
Notch signaling pathway	HDAC2, EP300, HDAC1, CREBBP, SNW1
